# *Guanxi* in an age of digitalization: toward assortation and value homophily in new tie-formation

**DOI:** 10.1186/s40711-022-00165-2

**Published:** 2022-07-02

**Authors:** Anson Au

**Affiliations:** Department of Applied Social Sciences, The Hong Kong Polytechnic University, Hung Hom, Kowloon, Hong Kong, People’s Republic of China

**Keywords:** *Guanxi*, Social networks, Tie-formation, Digitalization, Value homophily

## Abstract

How do people form personal ties? A consensus holds in sociological and social network scholarship that in-person networks are dominated by status homophily and that *guanxi* networks rely extensively on balance. This article argues that social networking sites (SNSs) reconceptualize the character of homophily and tie-formation altogether in *guanxi* networks. Drawing on 50 semi-structured interviews with Hong Kong youth from 2017 to 2020, this article examines how the technical capabilities of SNSs and principles of *guanxi* culture come together to erode status boundaries, create access to larger networks, and cause spillovers of information and tie strength. As a result, the basis of tie-formation in *guanxi* networks on SNSs shifts from balance to assortation and status homophily to value homophily. In this transformed calculus of tie-formation, two typologies of values rise to the fore: substantive values that reflect opinions and interests, as well as structural values that reflect networkability.

## Introduction

How do people form personal ties? A consensus holds in sociological and social network scholarship that ties are formed on the basis of one of two mechanisms: *balance* and *assortation*. In what is known as balance theory, the first mechanism of balance is when people form ties based on pressure toward reciprocity and mutuality in relations (Feld 1981, 1982; McFarland et al. 2014). With balance, people are inclined to network to avoid offending others, such as when a colleague introduces you to a friend in another department. The second mechanism of assortation filters the decision of networking based on personal preferences about the attributes of the actors involved. For instance, some people decide whom to network with based on homophily, how similar they are in terms of a social or status attribute (e.g., gender, education, McPherson et al. 2001), or heterophily, how different they are (Lin 1999).

In the Anglo-American sociological tradition, assortation has preoccupied the sociological imagination. In particular, much empirical and theoretical work on tie-building has limned the assortative mechanism of *homophily*. Largely defined as the preference for connecting with similar people (McPherson et al. 2001; Rivera et al. 2010), homophily has implications for the patterns and frequency with which people conduct exchanges in personal networks. Scott Feld (1981) posits that tie-formation results from the structure of a shared social context, around which people associate and which ultimately color the content of their associations. Here, Feld (1982) stresses the prevalence and formation of age homophilous friendships in various work settings. Research has since observed the prevalence of homophily in tie-building on both national, organizational, and personal levels (Kossinets and Watts 2009; Ladhari et al. 2020; McFarland et al. 2014).

However, little work has examined how homophily works to inform tie-formation behaviors on social networking sites (SNSs), which proliferates exponentially in social life. In particular, little work has taken up these lines of inquiry in China, where the rate of SNS proliferation is outpacing that of Western societies. According to the Pew Internet Research Center (Smith and Anderson 2018), SNSs have become increasingly popular social spaces for regular, intersectional, and multiplying interactions for civic participation, social interaction, and political participation (see also Gold 2012). Furthermore, it was China that led the world in 2020 as its number of SNS users (who used an SNS at least once a month) reached close to 930 million and is projected to rise to 1.13 billion by 2025, eclipsing even that of the United States (Statista 2020).

A distinguishing feature of SNSs is their ability to allow users to connect with others through online representations (profiles). This plasticity strips SNSs of the organizational structure on which much of homophilous networking in real life has been based (Feld 1981; McFarland et al. 2014), suggesting a transformation in the character of homophily online. Taking this as a point of departure, this article addresses these gaps in the literature by examining how SNSs transform tie-formation in Chinese guanxi.

In what follows, this article provides an overview of tie-formation mechanisms inhered in traditional *guanxi*. This article argues that the technical capabilities of SNSs and the cultural properties of *guanxi* networks combine to inspire users to build ties based on a new form of *assortation* founded on *values*. This argument is laid out in a theorization of the transformations to the architecture of social networks imputed by the rise of SNSs (the erosion of status boundaries, access to larger networks, and spillover of information and tie strength) and their subsequent invigoration of value homophily expressed in two types of values (substantive and structural values) that govern tie-formation in *guanxi*. This article then reviews the data and methods. Then, this article examines the cognitive interpretations and relational manifestations of the coupling of substantive values with the erosion of statuses, followed by that of structural values with superior network size and transitivity among interlocutors. This article concludes by reviewing the contributions of this research.

## Tie-formation in traditional Guanxi: balance and status homophily revisited

*Guanxi*, or the Chinese expression of social networks, is characterized by an orientation toward collectivist values. This collectivist orientation prefigures the form of individualized exchange and, by extension, the preferred tie-formation mechanism in personal networks. It means that individualized exchange remains a form of *cooperation* or *balance* (Chen et al. 2013; Liu and Mei 2015; Parnell 2005; Qi 2012). This can be understood as the social influence mechanism of solidarity within social networks (Marsden and Friedkin 1993). Like peer-group pressure, solidarity mandates abidance by cooperative norms in a larger social unit (Hwang 1987:947). In *guanxi*, people play to these pressures to change a contact’s attitudes or behaviors in conformity to their own (Emerson 1962:32).

Thus, people in *guanxi* are more likely to form ties based on *balance*, most of all in real-life settings, because they feel added pressure to reciprocate and avoid offending others, evinced by how ties embedded in organizations or groups are more likely to endure than non-embedded ones (Burt 2000; Bian 2018; McFarland et al. 2014). In real-life settings, people in *guanxi* primarily meet new alters in key social settings in the life course that tend to be highly bureaucratized and hierarchical, such as school or the workplace (Bedford 2011; Luo 2011). What results from the emphasis on balance in tie-formation in *guanxi* is an invigoration of role obligations. According to James Coleman (1990: 427–428), obligations of the role are when actors socially interact with others with respect to the “social structure of [their] positions in relation,” where their exchange is motivated by the “obligations and expectations [...] associated with their positions.” For instance, the presence of familial ties or different institutionalized roles in the same networks would produce a status difference—in which actor participation in exchange is driven by obligation to roles (ibid: 430; Barbalet 2021:370). Reflecting on the reliance on *guanxi* as an informal system of regulation in China, Jack Barbalet (2017: 2, italics added) observes the prevalence of such obligations of role in *guanxi*, which are rooted in “close personal monitoring, pervasive hierarchy-based dependence, and *role obligation*… the invasiveness of official powers and the high incidence of corruption, among other things… in Chinese society” (see also Barbalet 2018).

As a result of such obligations of role, people in *guanxi* rely on balance as a tie-formation mechanism to the effect of relying on status homophily, which is the tendency to connect with others who are similar in terms of statuses such as gender, ethnicity, and class (Ferrand et al. 1999; Laumann and Youm 1999; Lazarsfeld and Merton 1954; McPherson et al. 2001; McPherson and Smith-Lovin 1987). Empirical research on the general population in China shows a significant amount of status homophily in *guanxi*. Burt, Bian, and Opper find (2018) that *guanxi* entrepreneurial networks are predominantly male Han Chinese of similar ages and educational backgrounds. Blau et al. (1991) find that *guanxi* networks are even more homogeneous in gender and age than in the USA (see also Bian 2018).

Furthermore, Hwang (1987) observed that employees with *positive* relationships with their supervisors would take the initiative to *assist* individuals around their supervisors, as reciprocity for their debts to their supervisor and in the belief that the assisted would also help their supervisors. Extending this research, Liu and Wang (2013) conducted a survey of 280 employees and 280 direct supervisors in a Chinese organization to uncover how, conversely, *abusive* behavior by supervisors toward subordinates made subordinates *unwilling* to assist their supervisors, corroborating the significance of balance theory for understanding *guanxi* ties (see also Bu and Roy 2008; Yang and Wang 2011). This contrasts against organizational research in Western networks where, as Denise Rousseau (2001; Dabos & Rousseau, 2004) observes, workers honor their work obligations toward their managers, even when their psychological contracts go unfulfilled, such as when their employers do not provide adequate job satisfaction (see also Lee et al. 2011). Thus, balance lords over tie-formation in traditional *guanxi* as the pre-eminent networking mechanism, to the effect of abetting status homophily.

## Theorizing tie-formation in SNS networks: toward assortation and value homophily

How do people decide to build new ties in *guanxi* networks online? This article theorizes that the character of new tie-formation in *guanxi* is fundamentally transformed on SNSs. The ties studied in this article are predominantly weak in strength due to the nature of tie-formation itself: newly formed ties will not be strong, to begin with. The study of weak ties has been made famous with influential work based in North America and Europe, most impactfully Granovetter’s (1973) study of job matching in market economies that observed the utility of weak ties for providing diverse sources of job information and referrals that eventually percolated into a greater likelihood of securing a job. Much work has since transpired in extending the argument to the activation of ties in different job sectors and contexts (Bian and Ang 1997; Burt 2000; Lin 1999,2001).

Weak ties are also an important part of *guanxi* and engender different dynamics of interactions and cultural expectations than do weak ties in North America. Fei Xiaotong (1992[1947]), China’s first institutional sociologist, originally theorized in *From the Soil* that *guanxi* networks were ordered in concentric circles, with circles of ties rippling out from any given individual and where each circle was populated with ties of varying strengths. For Fei, those closest to the individual were traditionally kin who were strong ties, and those further out from the individual were non-kin who were weak ties. This stratification of tie strength was significant, Fei theorized, for different tie strengths engendered different obligations and utilization, where weak ties tended to service more instrumental needs, and strong ties were more used for expressive ones. Furthermore, ties were all beholden to the same orientation toward the collective in decision-making.

Empirical research has since corroborated the uniqueness and significance of weak ties in *guanxi* networks, in contradistinction to weak ties in North America and Europe. Yanjie Bian and Soon Ang (1997) observe that weak ties in East Asia reveal clear variation between tie strength and social resources compared to those in America. They find that the abstract and empirical foundations for *guanxi* to operate in Chinese societies are precisely the same orientation to the collective that Fei theorized, which takes precedence over the individual in *guanxi* across strong *and* weak ties alike. Put differently, the self is “identified, recognized, and evaluated in terms of one's relations to the groups and communities to which one belongs” (Bian and Ang 1997: 984). Reciprocal obligations extend to non-kin, weak ties as much as they do to kinship, strong ties, setting up a strong moral compulsion to reciprocate and bind people together if not in emotional closeness, then in cultural proximity in their shared values (Au 2020; Hwang 1987; Tian and Guo 2021; Yang 1994).

This, in essence, is what invigorates the importance and effectiveness of weak ties in *guanxi*. Unlike in North America, Bian and Ang (1997) find that even if weak ties are not as intensely emotive as strong ties, weak ties between job seekers and intermediaries are just as effective for matching individuals to jobs as strong ties. Li (2020) expands on this with a study of Chinese diasporic tourists visiting China, observing that weak ties between tourists and local communities help build the foundation for social mobility and connect with even more communities in the future. Indeed, weak ties at their core represent a kind of social capital that breeds more social capital, which has been essential for navigating social and work life in China’s unique labor market from its modern founding to the present (Bian 1997; Blau and Ruan 1990; Burt and Burzynska 2017; Nee and Opper 2012).

This article theorizes that digitalization is changing how new ties are being formed in *guanxi* as more and more networking transpires within the digital halls of SNSs: that the technical capabilities of SNSs and the cultural properties of *guanxi* networks combine to inspire users to build ties based on *assortation*—a powerfully stark departure for a networking culture that has historically prized balance. Just as important, this assortation is founded not on *statuses* but on *values*. Opposite to balance in traditional *guanxi*, assortation is the tendency to form ties based on individual preferences (McPherson et al. 2001). Incidentally, these preferences tend to home in on people’s values, including their beliefs and attitudes (Huckfeldt and Sprague 1995; Huston and Levinger 1978). Opposite to status homophily, value homophily is the tendency to form ties with others who are similar in terms of *values*. Assortation is coupled with value homophily, just as the balance is coupled with status homophily. In the US, for instance, Peter Marsden (1988) classically found that people exert choice in deciding whom to confide in *not* based on statuses like age but by similarities in values. Finkel et al. (2020) more recently find that adults exert this assortation to associate with similar political orientations, creating polarization we see at the group level and the national level. Oelberger (2018) observes that workplaces also foster a kind of value homophily, such as when international aid co-workers with similar working styles and meanings of work collaborate more closely and experience more enriching work relationships.

The roots of this shift toward assortation and value homophily as a tie-formation mechanism are theorized to be three interrelated network characteristics that arise from the unique form of SNSs.

(1) *Equality of statuses.* In in-person networks, statuses are an inescapable part of one’s network simply because the contexts (or foci) in which an ego meets alters are likely to be organizational in structure, and statuses are institutionalized in role differences. Demographic differences, such as gender, are also emphasized in in-person meetings based on appearances, cues, and social scripts that people follow (Kang 1997). Bedford (2011) finds that role-based status is among the most salient qualities that inspire confidence and compulsion to network for in-person *guanxi.* It may be tempting to suggest here that power differentials capture all motivations for interactions in *guanxi*, in a sweeping characterization of the instrumentalist nature of *guanxi* as Barbalet (2021) alludes. However, this is not an entirely accurate depiction of tie-formation in *guanxi*. First, a commonality emerges across all cases where role-based statuses impose on tie-formation: a tendency to form what Chen and Chen (2004) call an anticipatory base when interlocutors form a tie based on expectations primed not toward the past but the future. Put differently, power differentials are not an end but a means to an end. Moreover, second, the preoccupation with positional resources and power differentials is *subordinate* to another determinant of status in *guanxi* tie-formation: assessments of reputations by interpersonal networks based on *status-neutral etiquettes*. Individuals who fail to reciprocate or consistently reject requests of favors, for instance, risk being reputationally labeled rude, impolite, or even immorally uncivil (“*bu hui zuo ren* (不会做人)”), thus risk alienation from mainstream society regardless of status (Hsu 1996; Hwang 1987; Kinnison 2017).

On SNSs, tie-formation is becoming more status-neutral in these respects. First, information traditionally taken to be markers of status as positional resources is being de-emphasized in tie-formation on SNSs. This is aided by the design of SNSs that, first and foremost, curate user profiles based on the content they post, repost, and interact with, *not* backgrounds (Boyd 2006; Marwick and Boyd 2010). This portends significant implications for how people approach tie-formation on SNSs. To illustrate, although connections in Western SNS networks still show high levels of status homophily, where users prefer connecting with those similar in terms of age, education, race, religion, and other status characteristics (Centola 2011; Fiore and Donath 2005; Gu et al. 2014; Kwon et al. 2017), a loosely bound literature of *value* homophily is beginning to emerge. For example, a large-scale study of twelve million mentions among six million Twitter users identified the expression of values as the most important factor in users’ decisions to create new connections with someone (Šćepanović et al. 2017). Shorn of the barriers of geographical distance and blessed with an equalized ability to connect with someone of higher status directly, values have become important in online networks (Huber and Malhotra 2017).

Second, the status-neutral etiquettes that lubricate exchanges and inform people’s reputation among alters are being accentuated. A repertoire of grammatical logics and grammars of digital literacy endogenous to SNSs is newly emerging and fostering connections *across* demographic, generational, and social distances, such as the common appreciation for the meaning of social currencies (e.g., the number of *likes* and reposts) as signals of worth or the social construction of people’s profiles based on what they post (Au 2020). Similar results have been found in preliminary studies of SNS *guanxi* networks, such as Tian and Guo’s (2021) observation that there are cultural etiquettes governing interaction with alters on SNSs that are stripped of any reference to positional resources (e.g., using “okay” or “okie” instead of “ok” or “kk,” getting straight to the point when initiating a conversation rather than asking if they are available, not posting continuously on WeChat Moments), whereby failure to abide by them incurs the loss of reputation, regardless of role-based status.

(2) *Access to larger networks.* On SNSs, users typically have larger networks than they do in person. This is because SNSs ease the process through which connections can be made. Where in-person connections rely on symmetry, such as when two actors agree to be friends, SNS connections can be symmetric or asymmetric. Asymmetry occurs when connections are unrequited, such as when one actor one-sidedly “follows” another without reciprocity (Boyd 2006). SNSs further make visible information about a large number of fellow users, continually providing suggestions to users of new contacts to connect with. Often, this recommendation system draws from the networks of one’s alters. The fluidity with which ties are made on SNSs thus weakens group boundaries, resulting in greater transitivity (when indirect ties are formed, Haythornthwaite 2005; Romero and Kleinberg 2010; Ruppel et al. 2018).

(3) *Transitivity of information and tie strength.* Among in-person networks, information and emotional exchange are largely sequestered among one’s strong and weak ties (Aral and Alstyne 2011). Barbalet (2017) writes that guanxi networks are traditionally non-transitive because the opacity of information exchanged is valorized (see also King 1991). Exchanging information in strict confidence with select others creates a sense of exclusivity, which, in turn, acts as the basis for mutuality.

On SNS networks, however, information about users, including what and who they post, *like*, and follow, becomes publicized to strangers (Conroy et al. 2012). Like Western SNSs, there is thus a much greater quantity of social interactions exchanged among users on *guanxi* SNS networks than on in-person networks (Li and Peng 2019; Utz and Breuer 2017). This results in a spillover of tie strength, where ego can come to know about their non-ties, such as unfamiliar acquaintances, the same way they would know about strong or weak ties in in-person networks. As Au writes (2019; Au and Chew 2017), many users treat their personal Facebook as a kind of phonebook to keep the opportunity, but not an obligation, to contact unfamiliar acquaintances, where the decision to connect with one another is likable to the exchange of business cards. The constant expansion of one’s online network through the transitive and asymmetrical connection-building on SNSs thus makes for fertile ground to form ties in the future.

What kinds of values matter? This article theorizes that the technical design of SNSs’ format leads users to publicize information from which other users seek out cues of *substantive* values and *structural* values. Users then evaluate the reputation, trustworthiness, and networking prospects of non-ties based on similarities in these values.

(a) *Substantive values* refer to interests and opinions a user has. Sociologists have examined how people assign symbolic values to cultural interests for various purposes. Ann Swidler (1986) theorizes that people consume culture from myriad domains of everyday life and subsequently develop a repertoire of habits, skills, and styles people use to view the world and approach issues they encounter. Sometimes, this toolkit can be used to create basic social distinctions between people (Lamont and Molnár 2002). Other times, culture can be used to build bridges between people. Omar Lizardo (2006); Vaisey and Lizardo (2010) finds that popular culture consumption positively impacts the density of one’s network with weak ties. Building on this premise, we can observe that the myriad satellites hosted by SNSs representing special interests on SNSs (e.g., pages, groups, and blogs of interests, products, services, and public figures) serve as cultural artifacts for people to participate in (through *likes*) to express their own values and, by the same token, infer those of others’ (Au 2021). Furthermore, given how transitive and open information about users’ interests are on SNSs, the use of cultural interests as indicators of values should be magnified in tie-formation decisions.

(b) *Structural values* have to do with what can be called the networkability of a user. These values are called structural because they refer to the nominal conceptualization of an alter’s network structure. This is similar to the concept of network capital outlined by Wellman and Frank (2001), a kind of social capital dependent on one’s network reputation. Network capital considers whether one’s network is large enough, coordinated enough, or supportive enough to help when needed (ibid; Lin 2001:20). As Huggins et al. (2012: 208) note, network capital goes beyond conventional measures of social capital because it additionally considers “(1) the source of the capital; (2) the mechanisms through which the capital is created; (3) the objects of the capital; and (4) the impact of the capital.” What SNS users here look for in others’ structural values is a non-tie’s ability to create network capital, gauging how well they perform as a networker—whom they know and how they came to know them, which, in turn, reflect important information about their social lives, such as where and how they spend time. Esteban et al. (2021) use a study of social robots to demonstrate how the choice of whom to interact with is impinged upon preferences for more extroverted qualities. Using a survey experiment on friend-matching among university students, Huang et al. (2020) provide an important illustration of how people fasten themselves to social trait preferences (e.g., how reliable, generous, supportive, and independent an alter is) when networking with them, a pattern they argue to be exacerbated on SNSs. Rules deriving from *guanxi* culture come into play as benchmarks and guideposts for this performance, such as to inform etiquettes of respect, reciprocity, and reputation (Au 2021; Tian and Guo 2021).

Users not only monitor their own networking behaviors with these behavioral expectations, but they also impose them on non-ties pending connection. Thus, users may look to an alter’s network composition, how well networked a person is, and how affable they are to their ties as cues to infer whether a person is worth knowing, using past success as a barometer for future performance.

## Methodology

This article draws on two waves of semi-structured interviews with Hong Kong youth in 2017 to 2018 and 2019 to 2020 to investigate SNS use patterns across different platforms, including Facebook, Instagram, Twitter, WeChat, Weibo, and QQ. As Danah Boyd (2006) remarks, youth are digital natives who are the most active demographic category of SNS users and represent an ideal category to examine SNSs. Additionally, students as a demographic group are privy to the dynamics and demands of status differentials, evinced by studies of inequality between students of different status backgrounds (racial, ethnic, gender, and class), academic performance, and lifestyle habits (Armstrong et al. 2014; Park et al. 2014; Rankin and Reason 2005). The present sample thus provides a window to observe the future generational trajectory of ideations of status and values as they impinge upon decision-making for tie-formation in a rapidly advancing age of digitalization.

Analytically, this article focuses on individualized exchanges, which characterize the structure of exchanges flowing between linked actors in *guanxi*. Much work on the links between organizations in China, for instance, reveals a structure of (restricted) exchange similar to that between individual actors (Wu 2018). Interview questions were guided by themes about their different uses of SNSs, how they used them and to what end, how their interactions with others transpired and were structured online, contrasts between online and offline behavior, and reflections about their profiles and those of others.

The first wave recruited 24 participants from local Hong Kong universities. The second wave increased the sample size to 50 participants. Thirty-six were female, and 14 were male. Participants reported using Facebook and Instagram anywhere from 2.5 to 6 h a day in any given week, with an average of 3.5 h per day. Both waves were sampled with a non-random quota sampling scheme to capture the gender proportions of youth and university students in Hong Kong (The University of Hong Kong 2016). Sampling was motivated by what Mario Small (2009: 14) calls the creation of “a set of cases with particular characteristics that, rather than being ‘controlled away,’ should be understood, developed, and incorporated into her understanding of the cases at hand.” That is, this article sampled for depth—sampling as many people as possible within a specific, theoretically derived category (youth, in this study)—in order to build inferences about *processes* or the “essential linkage between two or more characteristics in terms of some explanatory schema” (Mitchell 1983: 199), such as behavioral schemas to do with networking. An additional merit of interviewing in two waves, furthermore, was its recreation of a sequential type of interviewing, where each additional case provided an “increasingly accurate understanding of the question at hand” until data saturation was achieved, whereby the same themes became recurrent among new interviewees (Small 2009: 24–25; Yin 2002).

Adopting a theoretically flexible approach to explore themes and patterns in masses of data, particularly in-depth interview data, qualitative thematic coding and a cross-comparative analysis of such themes are conducted (Attride-Stirling 2001). This approach finds similarities with cross-comparative coding frameworks native to grounded theory (Charmaz 2006). In the initial phase of open coding, the data were organized and labeled into different themes. In focused coding, commonalities were identified among those codes to formulate higher-level abstractions while recursively filtering the data to “determine the adequacy” of such codes (Charmaz 2006:57). Afterward, axial coding was conducted to reconstruct broader theorizations about networking practices and ideas by “[linking] categories with subcategories, and [asking] how they are related” (Charmaz 2006:61). The data was analyzed with both analytical induction (Katz 2001) and abductive analysis (Timmermans and Tavory 2012). Cases were compared within and across different themes to inductively and systematically depict the interpretive formation of social exchanges, simultaneously generating higher-level theorizations that make sense of the connections between *guanxi* tie-forming behaviors and technical capabilities of SNSs.

## The rise of substantive values: the erosion of statuses

For many users, SNSs were networking tools, where they connected with people who could essentially be characterized as non-ties or “stranger friends,” according to Kwan, a 20-year-old woman: acquaintances they were not close at present but with whom an SNS connection could keep a door open to potential tie creation in the future.

On what basis would users feel compelled to connect with someone on SNSs? For Kwan, the importance of values rose to the fore:

“I use the Internet as my way to connect with my friends or meet my new friends. I think we should treat it as a positive, like a new way to meet friends… my friends on Facebook, some of them are strangers, but after seeing what they *like*, we can be friends… like pages about beauty, *100 Mo* [a popular Hong Kong blog], Hong Kong life, unless something I do not really agree with.”

Treating her Facebook as a networking tool or collection of business cards, Kwan noted that she had connected with contacts on Facebook whom she did not know in real life and were essentially non-ties. This connection was intentional. Kwan strongly endorsed the possibility of building new ties with them by taking the initiative to interact with them, implicitly stressing that her intention for participating in SNS networking itself (“using the Internet”) was to build her network in a way that offline networks would not be able to. She emphasized, however, that her decision to create such new ties with others on SNS networks was contingent on the agreement of substantive values such as life interests.

According to Henry, a 21-year-old man, such substantive values were crucial to filter through in deciding whether or not to build a new tie with someone:

“Henry: It’s very rare for me to comment on things and interact with what acquaintances post. I’ll look at [their] views or opinions… about [social issues]… [then think about] what I think is correct about them and what I don’t think [is] right… this gives me some personal feelings. Then, I don’t comment unless I find they write about something really interesting, or something that I felt really deeply about. I’m very careful about my comments; they’re not as casual as *likes*.

Interviewer: Why’s that?

Henry: Because I’m afraid of people coming and picking a fight with [me].”

Couched in the strong terms of “right” and “wrong,” evident in Henry’s recollection was a search for symmetry in values, which he looked for in the content that an alter posted. Like Kwan, Henry felt that how well an Alter’s substantive values gave him “personal feelings”—an emotional sense of security and validation that there would be no conflict. He later provided an example of differing opinions on a celebrity infidelity scandal involving two high-profile Hong Kong musicians, with the majority siding with the wife, who was the victim, and a minority defending the unfaithful husband.

In a similar vein, Kwan recounted one of her most memorable exchanges on SNS and how it made her realize the significance of values in tie-building:

“Kwan: People always argue on the Internet. And there are some comments I think they are insulting. And because I think the thing that happens has its own reason. So, I will fight back against the negative or the argument that is untrue on Facebook [under comments on posts in my newsfeed]. And people will judge you back… basically, they don’t know you, and you don’t know them. It’s just like fighting a battle on the Internet, but they do not really exist in your own friends or your circle. So, I think it’s quite memorable because someone didn’t know you, but they judged you… they put their thoughts on you.”

This traumatic incident for Kwan was an alarm raised about the neutrality of statuses and the importance of similarity in values when interacting with the same non-ties she kept on her Facebook and with whom she contemplated building ties. Put differently, status-neutrality was a double-edged sword for Kwan—and is what gives strength to value homophily in networking on SNSs. On the one hand, people did not know Kwan just as she did not know them; this lent well to positive experiences of tie-formation, as she expressed freedom to reach across demographic and geographical boundaries to connect with someone. On the other hand, this same sense of freedom relieved people from caring about roles or statuses to “judge” others purely on the basis of their values and attack when their values disagreed. Both instances, as Kwan emphasized, reveal that it is the similarity in values alone that determines the quality and nature of new tie-formation on SNS networks.

According to Claire, a 20-year-old woman, the difference between values and —statuses is starker on SNS networks because of the typical visible information—and that which is not.

“Interviewer: How well do you think your Facebook profile represents who you are?

Claire: Facebook friends can look at my profile picture and the pages I *liked*. And my friends will understand me through these pages because these pages represent the issues that I’m interested in.

Interviewer: Okay, so they represent your interests.

Claire: Yes. [But for how well they can know me from my profile] I think people will know one’s personality only in reality. It’s so impossible on social media. Because not everything is there on social media.

Interviewer: So what kind of stuff isn’t there?

Claire: I seldom express my feelings online. Because I seldom talk so important things on Facebook. Because on Facebook, I tend to post my personal things and I do not want some strangers to know my personal issues, and I will talk these personal issues only to my good friends in reality.”

As Claire recounted, the use of SNSs is anchored in the circulation of social currencies such as *likes*—expressions of gratitude or endorsements that are afforded to one’s friends for tie-maintenance. However, these *likes* are also commonly extended to pages akin to websites, run by brands, businesses, public figures, and organizations and hosted on SNSs. The visibility of the pages that a user *likes* was, according to Claire, commonly taken to be a cue about their values.

Claire went further to stress that the observation of values based on such cues was all one could and should use to evaluate the networking prospects of friends on SNSs. Keeping in mind the public nature of SNSs, she refused to post much other than soundbites of her values, censoring what she thought was her personal issues. Here, the principle of exclusivity in *guanxi* culture uniquely emerges. For Claire, the decision to self-censor personal information was bound up by trust issues. Like how Western SNS users self-censor on sensitive topics for fear of backlash from strangers (Marwick and Boyd 2010), Chinese SNS users self-censor personal information for fear of it being poorly received by strangers (Au 2021), but also because of fear of offending friends they know as a possible breach of trust.

Users, therefore, were culturally inhibited as much as they were technically inhibited (in terms of the capabilities of SNSs) from discerning anything other than the values of other users, based on what they publicize. This sentiment was echoed by Kwan, who remarked,

“I don’t like arguments on the Internet, because I think… a particular post on the Internet, it's just like, a part of their life, it's not their whole person, it's not [the] whole of them. So I think that maybe the profile I walk into or… maybe just the things they post [would give me] the negative feelings to the person, but it doesn't mean that the person is a bad guy, or [that] he or she may really act in this way all the time.”

As Kwan alluded, she could not see anything but another user’s values from their profile and posts. Not only was the information that other SNS users presented scarce, but she was culturally primed to believe that what she saw were values; primed to cast the content she saw of other users into emotional valences (“negative feelings” or positive ones, conversely) that are reflective of values.

Notably absent in Kwan and Claire’s recollections was the importance of status. More than an alter’s education and class, they focused on perceiving the values embodied by an alter’s profile, posts, group memberships, and interest pages *liked* and the emotionality—a negative or positive impression—that they evoked. For both, as well as for Henry, SNSs opened up new circuits to *values* in the chemistry of tie-formation.

We can observe that SNSs made it easier to connect and made connections more fine-tuned to personal values. Unlike in real life, users did not need to spend much time interacting with an alter to discover what substantive values they had, instead of being able to infer this from the information that SNSs made public directly. This was, as Henry and Kwan alluded, useful to weed out potential conflicts down the road. In this way, users became *assortative* about whom they connected with.

The strong desire for symmetry in values reported by all users here marks a social psychological preference that can be called value homophily (Centola and Macy 2007; Ruef et al. 2003). McPherson and Smith-Lovin (1987) classically remark that homophily appears in relationships because its members feel more trusting, less uncertain, and more understood, and create a connection that reflects these qualities. In addition to identifying with these qualities, users reported feeling a greater sense of security (or “personal feelings,” in Henry’s words) from homogeneous values rather than heterogeneous ones.

This marks a significant departure from what we know of networking offline, in which status is paramount to networking because it predetermines the groups one can become a member of and the contexts in which one is regularly embedded (Ferrand et al. 1999; Centola and van de Rijt 2015). Using a natural experiment of students in America, for instance, McFarland et al. (2014) demonstrate that students tend to connect with demographically similar others, even when they are not obligated to by social structures in the school (see also McPherson and Smith-Lovin 1987).

Thus, although real-world connections often call upon statuses to judge an alter, users like Kwan, Henry, and Claire recounted that interactions on SNSs are shorn of restrictions like status. Alters can freely *like* and enter any page or group, after which the information is publicized for contacts to observe. Studies have found that this is true even of semi-private SNSs such as WeChat. Tian and Guo (2021, p. 21) find that Chinese users, for instance, appear eager to “[replicate] the patterns of everyday interactions among offline friend groups]…” which allows for this information flow when “users cannot see interactions among or information posted by friends of friends, among other technical affordances in the app,” such as to use “WeChat Moments” (*pengyou quan*).

The introduction of assortation by values or value homophily resulted in a novel kind of tie-making mechanism in *guanxi* on SNSs. Where in-person ties are more often formed amongst similar others along the lines of statuses, such as race or class (Laumann and Youm 1999; McPherson et al. 2001), *guanxi* ties fashioned on SNSs are ostensibly formed amongst similar others in terms of substantive values.

## The rise of structural values: greater network size and transitivity

As the previous section elaborated, users rely on cues of values to evaluate the reputation and trustworthiness of an alter. However, users also conceded that an alter’s profile only represented a sliver of someone’s entire persona, such that an alter’s substantive values—which they used to decide whether to trust a non-tie contact and ultimately build a new tie with—were not enough by themselves. To accommodate for this, users reported, they relied on another type of value to consider in the calculus of their tie-formation decisions: *structural* values.

Like how SNSs create cues for substantive values (information about the pages someone *likes*, the groups they join, the posts they make), they also create cues for structural ones. SNSs publicize each user’s list of contacts (called “friends” on Facebook, WeChat, QQ “following/followers” on Instagram, Weibo, and Twitter) and photographs that they are tagged in by others, through which users could visually observe how well networked an alter is.

Other empirical studies of SNS users in China observe that users work to curate a positive *image* of themselves on their profiles through the posts they make and the emotional energy they give off (Au and Chew 2017; Marwick and Boyd 2010). Here, users revealed that they scrutinized the images of others to determine whether to build a tie with them. After vetting an alter’s substantive values, users then looked for structural values that told how networkable a person was. In the words of George, a 24-year-old man,

“About maintaining the good impression, strangers don't know about me… [so] I show different personality to them. For people I know but not close friends, I *like* stuff [to be] very polite; I *liked* something that I didn’t really *like* but did so to show support… [not] from the heart. One of my friends helped me do something, and after this, I clicked like for her many times because I want to show others that I thanked her for her help with me doing something. But I didn't really care because she was selling insurance.”

Acts like George’s, done for the sake of “politeness,” reflect cultural etiquettes. Over time, users have created rules of behavior on SNSs in China that mandate they post and share content with “positive energy” on their public profiles, binding how well one observes these rules to their *mianzi* or reputation. However, users here revealed that they did not just participate in these etiquettes to satisfy their friends in dyadic exchange, but to cultivate a likeable, well-networked persona for non-ties as well. As George put it, doing this was necessary to “maintain [a] good impression” and project “a different personality to” strangers for the express purpose of being networkable.

Appearing networkable was both what users strove to emulate for non-ties who might consider building ties with them and what they themselves sought in non-ties with whom they were considering building ties. Zhang Yang, a 24-year-old woman, expands on this,

“I will take care of every detail of *me*. I think the pressure is from yourself. You like to be the perfect one on social media. And you want people; even if you are not familiar with them, you want them to *recognize* you… I [am] positive online, and I expect others to be too. If I see someone only posting negative comments all the time, I won’t feel like they’re a supportive person to connect with.”

For Zhang Yang, the structural values of users or their networkability depended on the emotional content of their comments (on others’ posts) as much as their posts (on their own profile). SNSs uniquely invigorate the emotional charge of one’s self-presentation in *guanxi*, where users self-censor their negative experiences and lionize their positive ones to curate a more positive profile. Here, we observe an extension of this to interpersonal relations: users sought evidence of positive expressive interactions by alters with others to weigh alters' structural values. Like George and Zhang Yang recounted, this too was part of gaining recognition for a networkable reputation by publicly appearing as someone supportive of their ties.

Kwan elaborates on this charismatic brand of recognition and the merits of appearing networkable as the core of successful tie-formation on SNSs: “Mutual friends can see what you post, and vice versa. [this way] I added and networked many friends that I basically don't know, who are mutual friends of one of my friends. If I see that someone has lots of friends and mutual friends, I will feel more open to connecting with them.”

Being able to see who someone is friends with and whether or not they are “polite,” users like George, Zhang Yang, and Kwan could observe how affable a non-tie was and evaluate how worthy they were of connection. In the same way, appearing affable also improved one’s own chances of expanding their networks, particularly among those with whom one had mutual friends—ties of one’s ties.

Josie, a 23-year-old woman, remarks on the pervasiveness of these mutual friends in *guanxi* networks when online, owing to the open channels of information on SNSs:

“Well, I really know that social media actually works in expanding social circles. Like in my school, when you see someone [on social media] now, you can easily find some mutual friends. Or when after I [hear of] someone's gossiping of another people, I will [come to] know of that person randomly. I think it's really hard to find a complete stranger due to social media…”

It was common, as Josie described, for information to travel fast on SNSs, whether users wanted to or otherwise. She highlighted the transitivity of information online, where profiles and information were readily available. As a result, she observed that even strangers both on and off her friends' list were not entirely strangers. Echoing George, Josie felt this added to her pressure to cultivate a networkable identity on SNSs.

Thus, the format of SNSs (all users’ networks being public and being able to visualize one’s mutual friends with a given alter) added to the cultural demands of *guanxi* (maintaining a well-networked reputation) to introduce newfound *transitivity* to online *guanxi* networks. In this way, larger network sizes (successful tie-formation) become both the cause and effect of transitivity. This dialectic, users recounted, is lubricated by structural values about someone’s networkability (as much as substantive ones).

As Golder and Yardi (2010) discovered in a web-based Twitter experiment, how well-connected someone is can be a catalyst for tie-formation. The better-connected someone is, the more likely they will build new ties with indirect ties. As users in this study corroborated, transitivity was associated with an increased desire to form a tie (Holland and Leinhardt 1970). Here, the collectivistic culture that defines *guanxi* catalyzes the tie-formation process further. In real life, *guanxi* ties are notoriously sticky: while creating a new tie can be difficult, maintaining a tie is comparatively easy.

The cultural desire for cooperation in *guanxi* culture works tremendously to valorize harmony in relations and repudiate confrontation. It is why, as Claire and Kwan recounted, members of *guanxi* networks are careful to avoid offending others: they work to preserve alters’ *mianzi* (reputation) in order to preserve their own (going so far, for instance, as to participate in nuanced exchanges of *likes* as gifts of *renqing*, Au 2021). Here, this means that the transitivity of information in visibly observing who is connected to who, and more importantly, the mutual friends that one has with any alter, creates a spillover of tie strength on SNSs.

After vetting structural and substantive values, users in *guanxi* networks on SNSs like Josie and Kwan become keen to connect with their alters’ alters and grow less likely to refuse invitations to connect with them out of fear of offending their alters, and so sullying their own networkability.

## Conclusion

Much of the social network literature preoccupied with homophily has limned *statuses* as the basis of tie-formation, which finds commonality with the significance of roles in ordering social interactions in traditional *guanxi* (Barbalet 2017). It is shown in the present article that among *guanxi* networks formed on SNSs, the basis of homophily in tie-formation shifts from statuses to *values* as the technical features and capabilities of SNSs refract configurations to the architecture of *guanxi* networks themselves. Information about one’s profile and ties is made visible to alters, which encourages scrutiny of one’s profile content more than status or demographical identifiers, larger network sizes, weaker geographical and group boundaries, and the transitivity of tie strength and information. The result is the invigoration of substantive values, in which users scrutinize resonances in personal interests and opinions with would-be ties prior to connecting, as well as structural values, in which users assess the networkability or network capital of would-be ties through the identities of any mutual friends and their track record of networking success, in informing tie-formation decisions.

The behavioral shifts in tie-formation documented in this article may raise questions about the nature of *guanxi*: to what extent are ties formulated through value similarities on SNSs still *guanxi*? However, the results have shown that the ties formed through value homophily still operate in the same way that ties formed through status homophily do. As George was keen to point out earlier, the cultural mandate of reciprocity held true in informing his interactions online as much as offline, where he felt compelled to offer support for an online alter (connected with based on structural values) who had gifted him with an earlier favor. Put differently, members of dyadic ties formed on the basis of value homophily in *guanxi* remain beholden to the same constraints (such as the demand for reciprocity and etiquettes of politeness) and opportunities (such as the ability to call on the tie for support) as those formed based on status homophily.

In an increasingly digitalized world, particularly in China, where SNS penetration and usage rates lead worldwide and are poised to surge, this article contributes to the literature on social networks and Chinese *guanxi* by identifying a prospective tectonic shift away from balance and status homophily (and their underlying obligations of role) that have long lorded over tie-formation in traditional *guanxi* networks toward novel nuances in assortation and value homophily (and obligations of exchange) orchestrated by the rise of SNSs. It is shown that SNSs are no longer mere tools for networking with traditional rules of *guanxi*, but social spaces of networking itself, where the cognitive and cultural rules of networking among Chinese users face transformation by the very design of SNSs themselves.

## Data Availability

Data supporting the findings in this study will not be shared for ethical concerns about protecting participants’ privacy.

## Abbreviaition

SNS(s): Social networking site(s)

## References

[CR1] Aral S, Van Alstyne M (2011). The diversity-bandwidth trade-off. American Journal of Sociology.

[CR2] Armstrong EA, Hamilton LT, Armstrong EM, Seeley JL (2014). “Good Girls” gender, social class, and slut discourse on campus. Social Psychology Quarterly.

[CR3] Attride-Stirling J (2001). Thematic networks: An analytic tool for qualitative research. Qualitative Research.

[CR4] Au A, Chew M (2017). How do you feel? Managing emotional reaction, conveyance, and detachment on Facebook and Instagram. Bulletin of Science, Technology & Society.

[CR5] Au A (2019). Thinking about cross-cultural differences in qualitative interviewing: Practices for more responsive and trusting encounters. The Qualitative Report.

[CR6] Au A (2020). Reconceptualizing the generation in a digital(izing) modernity: Digital media, social networking sites, and the flattening of generations. Journal for the Theory of Social Behaviour.

[CR7] Au A (2021). Guanxi 2.0: The exchange of likes in social networking sites. Information, Communication & Society.

[CR8] Barbalet J (2017). Dyadic characteristics of guanxi and their consequences. Journal for the Theory of Social Behaviour.

[CR9] Barbalet J (2018). Guanxi as social exchange: Emotions, power and corruption. Sociology.

[CR10] Barbalet, J. (2021). The analysis of Chinese rural society: Fei Xiaotong revisited. *Modern China*, 0097700419894921.

[CR11] Bedford O (2011). Guanxi-building in the workplace: A dynamic process model of working and backdoor guanxi. Journal of Business Ethics.

[CR12] Bian Y (2018). The prevalence and the increasing significance of guanxi. The China Quarterly.

[CR13] Bian Y, Ang S (1997). Guanxi networks and job mobility in China and Singapore. Social Forces.

[CR14] Blau PM, Ruan D (1990). Inequality of opportunity in urban China and America. Research in Social Stratification and Mobility.

[CR15] Blau PM, Ruan D, Ardelt M (1991). Interpersonal choice and networks in China. Social Forces.

[CR16] Burt RS, Burzynska K (2017). Chinese entrepreneurs, social networks, and guanxi. Management and Organization Review.

[CR17] Burt RS, Bian Y, Opper S (2018). More or less guanxi: Trust is 60% network context, 10% individual difference. Social Networks.

[CR18] Boyd, D. (2006). Friends, friendsters, and Myspace top 8: Writing community into being on social network sites. *First Monday 11*(2).

[CR19] Bu N, Roy JP (2008). Chinese managers' career success networks: The impact of key tie characteristics on structure and interaction practices. The International Journal of Human Resource Management.

[CR20] Burt RS (2000). The network structure of social capital. Research in Organizational Behavior.

[CR21] Centola D (2011). An experimental study of homophily in the adoption of health behavior. Science.

[CR22] Centola D, van de Rijt A (2015). Choosing your network: Social preferences in an online health community. Social Science & Medicine.

[CR23] Centola D, Macy M (2007). Complex contagions and the weakness of long ties. American Journal of Sociology.

[CR24] Charmaz K (2006). Constructing Grounded Theory: A Practical Guide Through Qualitative Analysis.

[CR25] Chen XP, Chen CC (2004). On the intricacies of the Chinese *guanxi*: A process model of *guanxi* development. Asia Pacific Journal of Management.

[CR26] Chen CC, Chen XP, Huang S (2013). Chinese guanxi: An integrative review and new directions for future research. Management and Organization Review.

[CR27] Chow IHS, Ng I (2004). The characteristics of Chinese personal ties (guanxi): Evidence from Hong Kong. Organization Studies.

[CR28] Coleman J (1990). Foundations of Social Theory.

[CR29] Conroy M, Feezell JT, Guerrero M (2012). Facebook and political engagement: A study of online political group membership and offline political engagement. Computers in Human Behavior.

[CR30] Dabos GE, Rousseau DM (2004). Mutuality and reciprocity in the psychological contracts of employees and employers. Journal of Applied Psychology.

[CR31] Emerson R (1962). Power-dependence relations. American Sociological Review.

[CR32] Esteban, P. G., Bagheri, E., Elprama, S. A., Jewell, C. I., Cao, H. L., De Beir, A., et al. (2021). Should I be Introvert or Extrovert? A Pairwise Robot Comparison Assessing the Perception of Personality-Based Social Robot Behaviors. *International Journal of Social Robotics,* 1–11.

[CR33] Farh JL, Tsui AS, Xin K, Cheng BS (1998). The influence of relational demography and guanxi: The Chinese case. Organization Science.

[CR34] Fei, X. (1992[1947]). *From the Soil: The Foundations of Chinese Society* (G. Hamilton & W. Zheng, Trans.). Berkeley, CA: University of California Press.

[CR35] Feld SL (1981). The focused organization of social ties. American Journal of Sociology.

[CR36] Feld SL (1982). Social structural determinants of similarity among associates. American Sociological Review.

[CR37] Ferrand A, Mounier L, Degenne A, Wellman B (1999). The Diversity of Personal Networks in France: Social Stratification and Relational Structures. Networks in the Global Village.

[CR38] Finkel EJ, Bail CA, Cikara M, Ditto PH, Iyengar S, Klar S (2020). Political sectarianism in America. Science.

[CR39] Fiore, A. T., & Donath, J. S. (2005, April). Homophily in online dating: when do you like someone like yourself?. In *CHI'05 extended abstracts on Human factors in computing systems* (pp.1371–1374).

[CR40] Gold M (2012). Debates in the digital humanities.

[CR41] Golder, S. A., & Yardi, S. (2010). Structural Predictors of Tie Formation in Twitter: Transitivity and Mutuality. In *2010 IEEE Second International Conference on Social Computing.*

[CR42] Granovetter MS (1973). The strength of weak ties. American Journal of Sociology.

[CR43] Gu B, Konana P, Raghunathan R, Chen HM (2014). Research Note—The Allure of Homophily in Social Media: Evidence from Investor Responses on Virtual Communities. Information Systems Research.

[CR44] Haythornthwaite C (2005). Social networks and Internet connectivity effects. Information, Communication & Society.

[CR45] Holland PW, Leinhardt S (1970). A method for detecting structure in sociometric data. American Journal of Sociology.

[CR46] Hsu SC (1996). “Face”: An Ethnographic Study of Chinese Social Behavior.

[CR47] Huang SA, Ledgerwood A, Eastwick PW (2020). How do ideal friend preferences and interaction context affect friendship formation? Evidence for a domain-general relationship initiation process. Social Psychological and Personality Science.

[CR48] Huber GA, Malhotra N (2017). Political homophily in social relationships: Evidence from online dating behavior. The Journal of Politics.

[CR49] Huckfeldt R, Sprague J (1995). Citizens, Politics and Social Communication: Information and Influence in an Election Campaign.

[CR50] Huggins R, Johnston A, Thompson P (2012). Network capital, social capital and knowledge flow: How the nature of inter-organizational networks impacts on innovation. Industry and Innovation.

[CR51] Huston TL, Levinger G (1978). Interpersonal attraction and relationships. Annual Review of Psychology.

[CR52] Hwang KK (1987). Face and Favor: The Chinese Power Game. American Journal of Sociology.

[CR53] Kang ME (1997). The portrayal of women’s images in magazine advertisements: Goffman’s gender analysis revisited. Sex Roles.

[CR54] Katz, J. (2001). Analytical Induction. In N. Smelser & P. Baltes (Eds.), International encyclopedia of the social and behavioral sciences (Vol. 1, pp. 480–484). Elsevier.

[CR55] King AYC (1991). Kuan-hsi and network building: A sociological interpretation. Daedalus.

[CR56] Kinnison LQ (2017). Power, integrity, and mask—An attempt to disentangle the ChineseFace concept. Journal of Pragmatics.

[CR57] Kossinets G, Watts DJ (2009). Origins of homophily in an evolving social network. American Journal of Sociology.

[CR58] Kwon HE, Oh W, Kim T (2017). Platform structures, homing preferences, and homophilous propensities in online social networks. Journal of Management Information Systems.

[CR59] Lamont M, Molnár V (2002). The study of boundaries in the social sciences. Annual Review of Sociology.

[CR60] Ladhari R, Massa E, Skandrani H (2020). YouTube vloggers’ popularity and influence: The roles of homophily, emotional attachment, and expertise. Journal of Retailing and Consumer Services.

[CR61] Laumann E, Youm Y (1999). Racial/ethnic group differences in the prevalence of sexually transmitted diseases in the United States: A network explanation. Sexually Transmitted Diseases.

[CR62] Lee C, Liu J, Rousseau DM, Hui C, Chen ZX (2011). Inducements, contributions, and fulfillment in new employee psychological contracts. Human Resource Management.

[CR63] Li L, Peng W (2019). Transitioning through social media: International students' SNS use, perceived social support, and acculturative stress. Computers in Human Behavior.

[CR64] Li TE (2020). Guanxi or weak ties? Exploring Chinese diaspora tourists’ engagements in social capital building. Current Issues in Tourism.

[CR65] Lin N (1999). Social networks and status attainment. Annual Review of Sociology.

[CR66] Lin N (2001). Social Capital: A Theory of Social Structure and Action.

[CR67] Liu L, Mei S (2015). How can an indigenous concept enter the international academic circle: The case of *guanxi*. Scientometrics.

[CR68] Liu XY, Wang J (2013). Abusive supervision and organizational citizenship behaviour: Is supervisor–subordinate guanxi a mediator?. The International Journal of Human Resource Management.

[CR69] Lizardo O (2006). How cultural tastes shape personal networks. American Sociological Review.

[CR70] Luo JD (2011). Guanxi revisited: An exploratory study of familiar ties in a Chinese workplace. Management and Organization Review.

[CR71] Marsden PV (1988). Homogeneity in confiding relations. Social Networks.

[CR72] Marsden PV, Friedkin NE (1993). Network studies of social influence. Sociological Methods & Research.

[CR73] Marwick AE, Boyd, D (2010). I tweet honestly, I tweet passionately: Twitter users, context collapse, and the imagined audience. New Media & Society.

[CR74] McFarland DA, Moody J, Diehl D, Smith JA, Thomas RJ (2014). Network ecology and adolescent social structure. American Sociological Review.

[CR75] McPherson JM, Smith-Lovin L (1987). Homophily in voluntary organizations: Status distance and the composition of face-to-face groups. American Sociological Review.

[CR76] McPherson M, Smith-Lovin L, Cook JM (2001). Birds of a feather: Homophily in social networks. Annual Review of Sociology.

[CR77] Mitchell JC (1983). Case and situation analysis. Sociological Review.

[CR78] Nee V, Opper S (2012). Capitalism From Below.

[CR79] Oelberger CR (2019). The dark side of deeply meaningful work: Work-relationship turmoil and the moderating role of occupational value homophily. Journal of Management Studies.

[CR80] Park JJ, Denson N, Bowman NA (2013). Does socioeconomic diversity make a difference? Examining the effects of racial and socioeconomic diversity on the campus climate for diversity. American Educational Research Journal.

[CR81] Parnell MF (2005). Chinese business guanxi: An organization or non-organization. Journal of Organisational Transformation & Social Change.

[CR82] Qi X (2012). A case study of globalized knowledge flows: Guanxi in social science and management theory. International Sociology.

[CR83] Rankin SR, Reason RD (2005). Differing perceptions: How students of color and white students perceive campus climate for underrepresented groups. Journal of College Student Development.

[CR84] Rivera MT, Soderstrom SB, Uzzi B (2010). Dynamics of dyads in social networks: Assortative, relational, and proximity mechanisms. Annual Review of Sociology.

[CR85] Romero, D. M. & Kleinberg, J. (2010). The directed closure process in information networks with an analysis of link formation on twitter. In *International Conference on Weblogs and Social Media (ICWSM).*

[CR86] Rousseau DM (2001). Schema, promise and mutuality: The building blocks of the psychological contract. Journal of Occupational and Organizational Psychology.

[CR87] Ruef M, Aldrich HE, Carter NM (2003). The structure of founding teams: Homophily, strong ties, and isolation among US entrepreneurs. American Sociological Review.

[CR88] Ruppel EK, Burke TJ, Cherney MR (2018). Channel complementarity and multiplexity in long-distance friends’ patterns of communication technology use. New Media & Society.

[CR89] Šćepanović S, Mishkovski I, Gonçalves B, Nguyen T, Hui P (2017). Semantic homophily in online communication: Evidence from twitter. Online Social Networks and Media.

[CR90] Simmel G (1955). Conflict and the Web of Group Affiliations.

[CR91] Small ML (2009). How many cases do I need? On science and the logic of case selection in field-based research. Ethnography.

[CR92] Smith A, Anderson M (2018). Social media use in 2018. Pew Research Center.

[CR93] Song F, Cadsby SB, Bi Y (2012). Trust, reciprocity, and guanxi in China: An experimental investigation. Management and Organization Review.

[CR94] Statista.  (2020). Number of Social Network Users Worldwide from 2017 to 2025.

[CR95] Swidler A (1986). Culture in action: Symbols and strategies. American Sociological Review.

[CR96] Tian, X. & Guo, Y. (2021). An Online Acquaintance Community: The Emergence of Chinese Virtual Civility. *Symbolic Interaction*. Published Online ahead of Print. 10.1002/symb.537

[CR97] Timmermans S, Tavory I (2012). Theory construction in qualitative research from grounded theory to abductive analysis. Sociological Theory.

[CR98] Utz S, Breuer J (2017). The relationship between use of social network sites, online social support, and well-being: Results from a six-wave longitudinal study. Journal of Media Psychology: Theories, Methods, and Applications.

[CR99] Vaisey S, Lizardo O (2010). Can cultural worldviews influence network composition?. Social Forces.

[CR100] Van den Bulte C, Lilien GL (2001). Medical innovation revisited: Social contagion versus marketing effort. American Journal of Sociology.

[CR101] Wellman B, Wortley S (1990). Different strokes from different folks: Community ties and social support. American Journal of Sociology.

[CR102] Yang M (1994). Gifts, Favors and Banquets: The Art of Social Relationship in China.

[CR103] Yang Z, Wang CL (2011). Guanxi as a governance mechanism in business markets: Its characteristics, relevant theories, and future research directions. Industrial Marketing Management.

[CR104] Yin R (2002). Case Study Research.

